# Efficacy of BCG Vaccination against COVID-19: Systematic Review and Meta-Analysis of Randomized Controlled Trials

**DOI:** 10.3390/jcm12031154

**Published:** 2023-02-01

**Authors:** Jiayu Wen, Quanxian Liu, Daoyan Tang, Jian-Qing He

**Affiliations:** Department of Respiratory and Critical Care Medicine, West China Hospital, Sichuan University, Chengdu 610041, China

**Keywords:** COVID-19, BCG, vaccine, trained immunity, SARS-CoV-2

## Abstract

Beneficial off-target effects of the Bacillus Calmette-Guérin (BCG) vaccination might offer general protection from respiratory tract infections. We conducted a systematic review and meta-analysis of published randomized controlled trials (RCTs) to ascertain BCG vaccination effectiveness against COVID-19. We looked up English RCTs from 1 January 2019 to 15 November 2022 in Embase, the Cochrane Library, and the Web of Science in this systematic review and meta-analysis. Nine RCTs, including 7963 participants, were included. The infection rate of COVID-19 was not decreased in people who were vaccinated with BCG (OR, 0.96; 95% CI, 0.82–1.13; I^2^ = 4%), and the BCG vaccination group did not have decreased COVID-19 related-hospitalization (OR, 0.66; 95% CI, 0.37–1.18; I^2^ = 42%), admission to the ICU (OR, 0.25; 95% CI, 0.05–1.18; I^2^ = 0%), and mortality (OR, 0.64; 95% CI, 0.17–2.44; I^2^ = 0%) compared with the control group. There is not sufficient evidence to support the use of BCG vaccination in the prevention of COVID-19 infection and severe COVID-19 and avoid overstating the role of BCG vaccination leading to its misuse.

## 1. Introduction

Since the end of 2019, a new virus known as severe acute respiratory syndrome coronavirus-2 (SARS-CoV-2) has been sweeping the globe, triggering coronavirus disease 2019 (COVID-19).

Early in the 20th century, the Bacillus Calmette-Guerin (BCG) vaccine was created to prevent tuberculosis [1]. Furthermore, BCG has beneficial off-target (i.e., non-specific or heterologous) effects, which are becoming increasingly recognized. For example, BCG vaccination is related to lower all-cause mortality in babies and all-caused respiratory disease morbidity in the elderly, as well it can protect against human experimental models of yellow fever in healthy adults [2,3,4]. Due to the off-target effects of the BCG vaccine, it can alter immunologic set points via heterologous T-cell immunity or reprogramming of innate immune cells [3,5,6,7]. BCG vaccination might be more beneficial in conditions where such viral variations are abundant by enhancing antiviral host defense in an antigen-independent manner compared with the COVID-19 vaccine [8].

In current studies, it is controversial whether the BCG vaccine can be used against COVID-19 [9,10,11,12,13,14,15,16,17]. For example, Berg et al. found BCG vaccination could reduce mortality and morbidity rates by comparing the COVID-19 infection and death records from countries with a national BCG vaccination program to countries without [10]. However, the study of Arlehamn et al. did not support the assertion that BCG could reduce COVID-19 mortality when using updated mortality data [17]. Furthermore, Pépin et al. demonstrated that BCG vaccination did not reduce COVID-19 infection, hospitalization, or mortality rates [9].

To determine the efficacy of BCG vaccination against COVID-19, we conducted a systematic review and meta-analysis of randomized controlled trials.

## 2. Materials and Methods

### 2.1. Data Sources and Searches

The review protocol was registered in the PROSPERO International Prospective Register of Systematic Reviews (registration number: CRD42022339994) and conducted according to the preferred reporting items for systematic reviews and meta-analysis statements [18]. We searched PubMed, Embase, Cochrane Library, and Web of Science for relevant literature from 1 January 2019 to 15 November 2022. The search included the terms (“COVID-19” OR “SARS-CoV-2”) AND (“BCG Vaccine” OR “Mycobacterium Bovis”); the detailed search approach is outlined in the Supplementary Material (Supplementary Material: Appendix S1). In addition, manual backward searches of references from included studies were also conducted. The result was transferred to EndNote X9 for additional evaluation.

### 2.2. Selection of Studies

Inclusion criteria: (1) Population (P): Participants were adults (aged 18 years or over) who tested positive for COVID-19 by PCR or rapid antigen. There were no restrictions on gender, race, ethnicity, or geographical distribution; (2) Intervention (I): any strain BCG at any dosage (3) Control (C): placebo or no treatment; (4) Outcome (O): primary outcomes: The primary outcome is the incidence of COVID-19. The secondary outcomes were hospitalization, admission to the intensive care units (ICU)and mortality of COVID-19, and the risk of adverse events (AEs). All outcomes were followed for the longest follow-up periods for each one. Last, Randomized controlled trials (RCTs) in English published were included.

We excluded observational studies, research protocols, reviews, news, case reports, abstracts from conferences, unpublished publications accessible on preprint services, animal studies, in vivo experiments, animal research, and studies that did not test for COVID-19 infections.

### 2.3. Study Selection and Data Extraction

Two writers (JYW and QXL) independently read the titles and abstracts of the papers to find those that satisfied the inclusion criteria and extracted data. In the absence of unanimity, a third reviewer (JQH) was consulted. Information was extracted into an Excel spreadsheet. We extracted the following information: (1) first author; (2) year of publication; (3) participant characteristics; (4) intervention/exposure and control; (5) the type of COVID-19 diagnosis; and (6) data regarding BCG vaccination efficacy and risk of AEs as study outcomes were available.

### 2.4. Evaluation of the Risk of Bias and Quality

The risk of bias in RCTs was evaluated using RoB 2, a redesigned instrument for evaluating the risk of bias in randomized trials. RoB 2 was comprised of the following five domains: (1) randomization process, (2) deviations from intended interventions, (3) missing outcome data, (4) measurement of the outcome, and (5) selection of the reported result. Each of these domains is assigned a risk level that ranges from “High risk” to “Some concerns” to “Low risk” [19]. To assess the quality of evidence, GRADE (Grading of Recommendations Assessment, Development, and Evaluation) recommendations were used and were classified into “high,” “medium,” “low,” and “very low,” which were based on the risk of bias, consistency, directness, precision, and publication bias [20]. When one of the above criteria was not met, the assigned quality level was lowered.

The evaluations were carried out independently by two investigators (JYW and DYT). Disputes between the two investigators were resolved through discussion or by the third investigator (QXL).

### 2.5. Statistical Analysis

Continuous variables were reported as the mean and standard derivation (SD), while dichotomous variables were reported as the frequency and proportion. The effect size was summarized as odds ratios (ORs) with confidence intervals (CIs), which were displayed in forest plots. Statistical heterogeneity among studies was calculated by χ^2^-based Q test and I^2^ statistics, with I^2^ > 50% considered statistically significant. Values of 25%, 50%, and 75% were used as cutoff points for low, medium, and high inconsistency [21]. Pooled outcomes with 95% confidence intervals (95% CIs) were calculated using the random-effect model if I^2^ > 50%; otherwise, the fixed-effect model was used.

Sensitivity analyses were conducted by sequentially deleting studies to examine the robustness of the aggregated data. In addition, preplanned subgroup analyses were based on the strains in the vaccine. Each BCG strain belonged to a different subgroup that consisted of at least two studies.

Egger’s test and funnel plots were used to assess the presence of publication biases. Potential missing studies were imputed using the trim-and-fill method if publication bias was suspected (*p* < 0.05).

All statistical analyses were carried out using Stata (Version 16; StataCorp, College Station, TX, USA) and Review Manager, Version 5.4.1 (Cochrane Collaboration).

## 3. Results

### 3.1. Search Results and Study Characteristics

**Figure 1:**
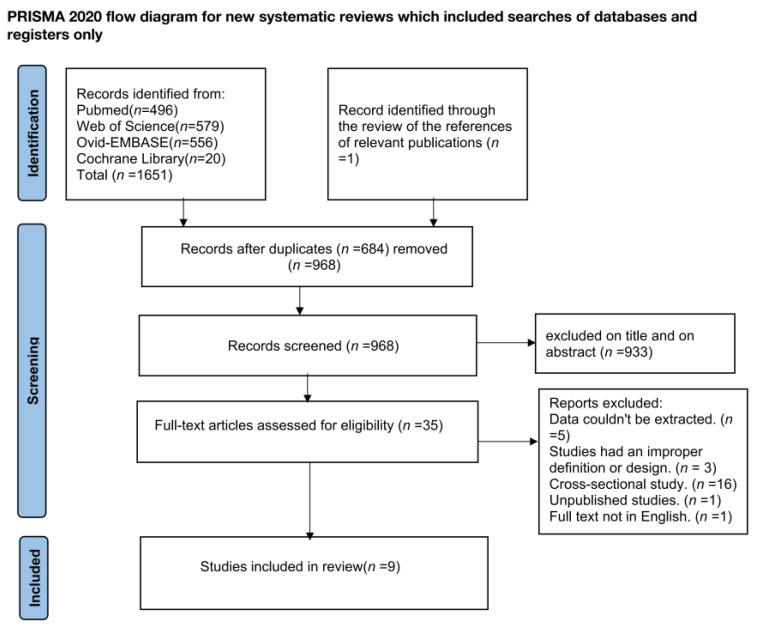
The detailed flow chart of the Literature search.

The search approach uncovered 1652 potentially relevant studies, of which 684 were omitted owing to duplication. Among the remaining 968 studies, 933 were excluded due to their titles or abstracts. Therefore, 35 studies were eligible for full-text review, and 26 studies were excluded for the following reasons: unavailable data (*n* = 5), improper definition and design (*n* = 3), observational studies (*n* = 16), unpublished study (*n* = 1), and full-text not in English (*n* = 1). Eventually, the current study consisted of a total of nine randomized controlled trials that enrolled 7963 participants and were published in 2022 [22,23,24,25,26,27,28,29,30] (Figure 1).

In all of the studies that were included, the experimental and control groups were well-balanced in terms of baseline demographics, such as age and gender ratio [22,23,24,25,26,27,28,29,30]. Seven of the nine included studies reported that the precise BCG vaccination history [22,23,24,25,26,27,29] and comorbidities [23,24,25,26,27,28,29] were balanced between the two groups, with no statistically significant difference. However, two lacked information on the study participants’ precise BCG vaccination history [28,30] and comorbidities [22,30]. All participants were given intradermal BCG.

Among the nine RCTs, five were multicenter studies (Poland [22], Greece [28], the Netherlands [23,26], and Germany [30]), and four were single-center studies (Brazil [24], South Africa [29], the United States [25] and India [27]).

Three studies included data on older adults [26,28,30], one on patients with type 1 diabetes [25], and the remaining four studies enrolled healthcare workers [22,23,24,29]. For the diagnosis of COVID-19, seven studies solely relied on a positive PCR test [22,23,26,27,28,29,30], one study depended on both a positive PCR test and symptoms [25], and another study relied on either the positive PCR test or rapid antigen test [24]. Moreover, the sample size ranged from 131 to 2015, and the follow-up time spanned from 3 to 15 months. The detailed information is shown in Table 1.

### 3.2. Primary Outcomes

#### The Rate of Infection of COVID-19

**Figure 2:**
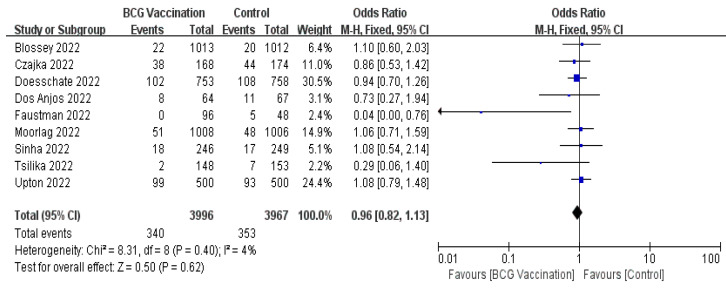
Forrest plot of the odds ratio of the incidence of COVID-19 between the BCG vaccination group and the control group.

A total of nine RCTs [22,23,24,25,26,27,28,29,30], including 7963 participants, evaluated BCG vaccination effectiveness against COVID-19. The results showed that the incidence of COVID-19 infection was not significantly decreased in people who were vaccinated with BCG using the fixed-effect model (OR, 0.96; 95% CI, 0.82–1.13; Figure 2). There was low heterogeneity among all included studies of COVID-19 (I^2^ = 4%, *p* = 0.4; Figure 2).

**Figure 3:**
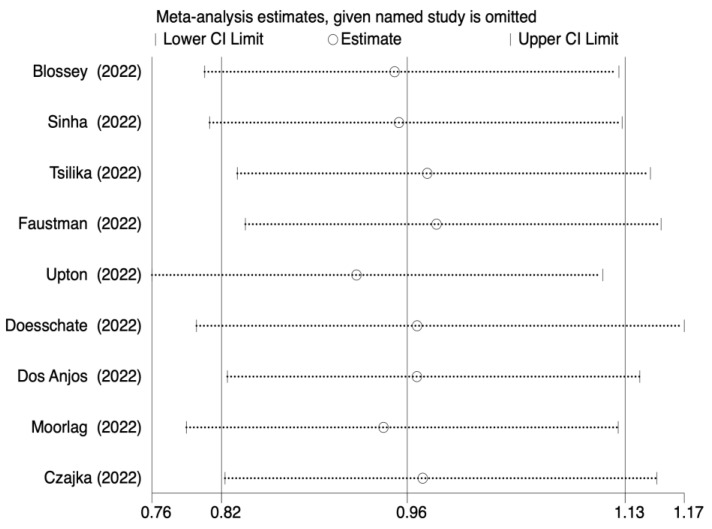
Sensitivity analysis result of the association between BCG vaccination and incidence of COVID-19.

The leave-one-out sensitivity analysis also indicated that individual research studies did not affect the pooled incidence rate (Figure 3). An analysis of the subgroup that had been preplanned based on the BCG strain was carried out. The results also did not change in the BCG Moscow group (OR, 0.81; 95% CI, 0.48–1.35; I^2^ = 15%) and Danish strain 1331 group (OR, 1.02; 95% CI, 0.84–1.23; I^2^ = 0%). There was no intergroup heterogeneity in the two groups (I^2^ = 15%, *p* = 0.31; I^2^ = 0%, *p* = 0.80, respectively) (Supplementary Material: Appendix S2 Figure SA1).

### 3.3. Secondary Outcomes

#### 3.3.1. The COVID-19-Related Hospitalization

**Figure 4:**
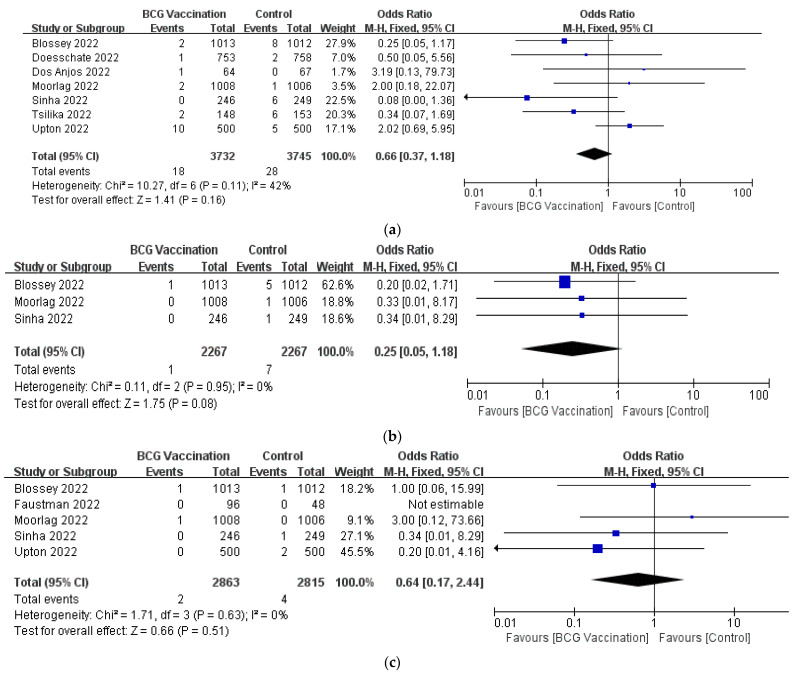
Forrest plot of the odds ratio of the (**a**) hospitalization; (**b**) admission to the ICU; (**c**) mortality of COVID-19 between the BCG vaccination group and the control group.

All seven studies reported COVID-19 hospitalizations [23,24,26,27,28,29,30]. According to the fixed-effect model, there was no statistically significant difference between the BCG vaccination group and the control group (OR, 0.66; 95% CI, 0.37–1.18; I^2^ = 42%) (Figure 4a).

#### 3.3.2. The COVID-19-Related Admission to the ICU

There was no significant difference observed in COVID-19-related admission to the ICU between participants vaccinated or not vaccinated with BCG of three RCTs (OR, 0.25; 95% CI, 0.05–1.18; I^2^ = 0%) (Figure 4b) [26,27,30].

#### 3.3.3. The COVID-19-Related Mortalitys

A fixed effect meta-analysis of five trials showed no significant difference between the BCG vaccination and control group in terms of COVID-19-related mortality (OR, 0.64; 95% CI, 0.17–2.24; I^2^ = 0%) (Figure 4c) [25,26,27,29,30].

#### 3.3.4. The Safety of BCG Vaccination

**Figure 5:**
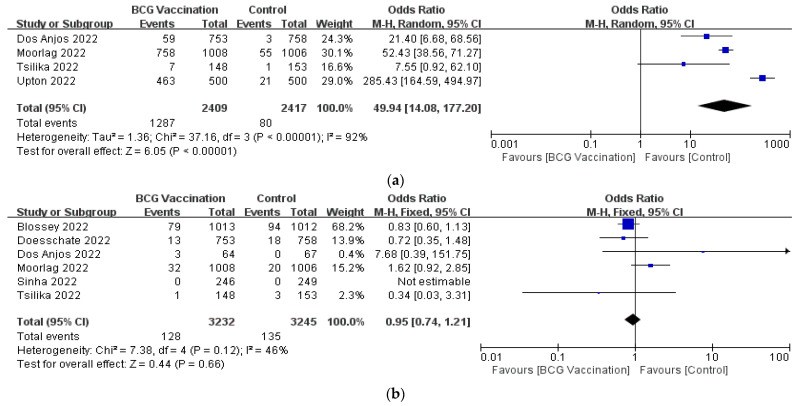
Forrest plot of the odds ratio of the (**a**) local injection response; (**b**) serious AEs between the BCG vaccination group and the control group.

In terms of specific AEs, we found local injection reactions were more common in the BCG group compared to the control group in four RCTs (OR, 49.94; 95% CI, 14.08–177.2) and there was high heterogeneity between the studies (*p* < 0.00001; I^2^ = 92%) [24,26,28,29] (Figure 5a). The subgroup analyses according to the BCG strain for both the BCG Moscow (OR, 17.88; 95% CI, 6.49–49.25; I^2^ = 0%) and BCG Danish 1331 groups (OR, 76.23; 95% CI, 58.42–99.48; I^2^ = 96%) showed similar results, and there was still substantial heterogeneity in the Danish strain 1331 group (Appendix S2 Figure SA2). Six RCTs reported serious AEs, but there were no discernible differences between them (OR, 0.95; 95%CI, 0.74–1.21; I^2^ = 46%) [2,23,24,26,27,28] (Figure 5b).

### 3.4. Risk of Bias and Publication Bias

**Figure 6:**
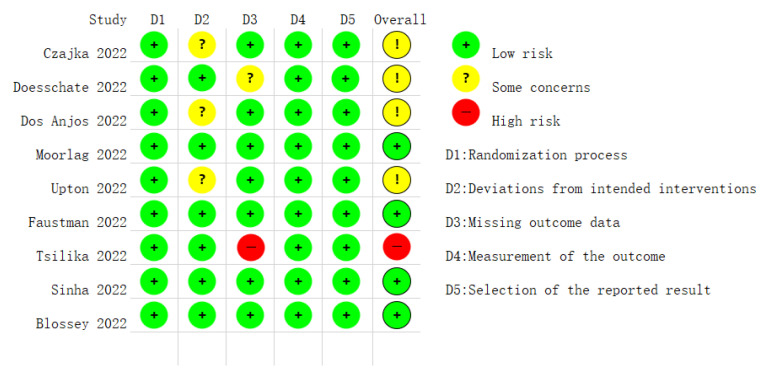
Quality assessment of the risk of bias in the studies.

For nine RCTs, four were judged to have low bias concerns [26,27,29,30], while four were judged to have some concerns of bias regarding “deviations from intended interventions” and “missing outcome data” [22,23,24,29]. In addition, one study had a high-risk bias for missing outcome data [28]. The comprehensive evaluation of bias risk is given in Figure 6.

**Figure 7:**
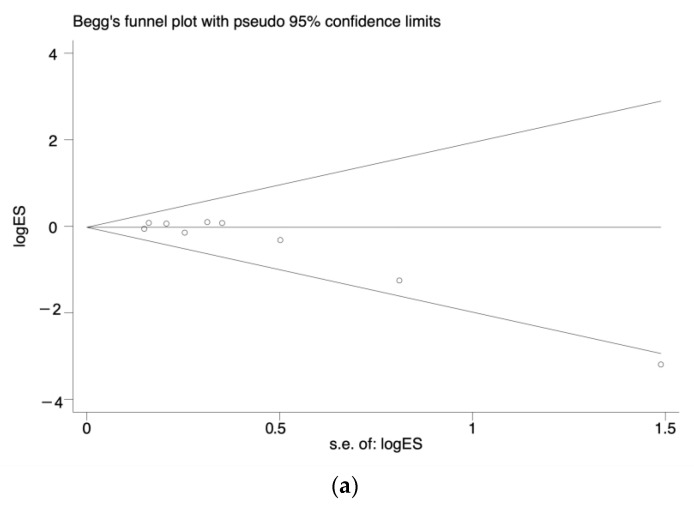

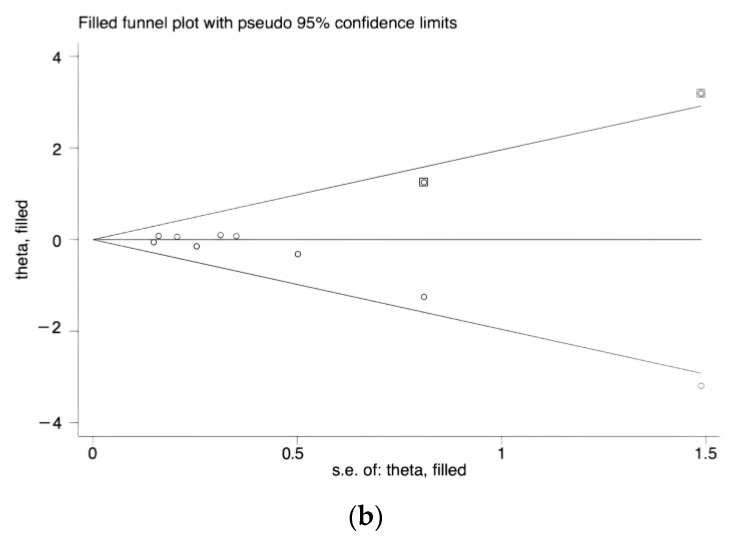
Publication bias analysis of the infection of COVID-19 between the BCG vaccination group and control: (**a**) The Funnel plot. (**b**) Trim and fill analysis.

For the primary outcome, the asymmetry shown by visual inspection of the funnel plot (Figure 7a) was statistically significant, and Egger’s test was similar (*p* = 0.017). To further verify its influence on results, trim-and-fill adjustment was performed. Adding two studies had no significant impact on the adjusted results of our primary outcome (fixed effects model, OR, 1.00; 95% CI, 0.85–1.17) or (random effects model, OR, 1.00; 95% CI, 0.79–1.26) (Figure 7b). For secondary outcomes, which included COVID-19-related hospitalization, COVID-19-related mortality, and serious AEs, there was no evidence of publishing biases. The funnel plots were displayed in the Supplementary Material: Appendix S2 Figure SA3. No publication bias tests were performed for the COVID-19-related ICU and local injection responses since there were not enough trials.

### 3.5. Grade Evaluation

Table 2 displayed GRADE assessments of the quality of evidence for all outcomes. The primary outcome was evaluated as ‘moderate’ due to publication bias.

## 4. Discussion

In this study, we assessed the effectiveness of BCG vaccination against COVID-19. This meta-analysis demonstrated that BCG vaccination did not significantly provide prevention for COVID-19. The following data provided support for this conclusion. First, we discovered that, compared to the control group, those who received the BCG vaccination had no significantly lower incidence of COVID-19 infection (OR, 0.97; 95% CI, 0.84–1.11) with low heterogeneity (I^2^ = 4%). Second, the result did not change in the leave-one-out sensitivity analysis. Furthermore, the BCG vaccine was first introduced in 1921. During their passage, BCG strains accumulated genomic alterations leading to the emergence of several substrains. It has been discovered that the immune response amplitude varies according to the strain [31,32]. We analyzed subgroup analyses based on BCG strain; the results were similar.

In addition, our study also discovered BCG vaccination could not prevent severe COVID-19 in terms of COVID-19-related hospitalization (OR, 0.66; 95% CI, 0.37–1.18; I^2^ = 42%), admission to the ICU (OR, 0.25; 95% CI, 0.05–1.18; I^2^ = 0%), and mortality (OR, 0.64; 95% CI, 0.17–2.44; I^2^ = 0%). In addition, we found the BCG vaccination group experienced more local injection response (OR, 49.94; 95% CI, 14.08–177.2; I^2^ = 92%) compared with the control group, but the serious AEs were similar (OR, 0.95; 95%CI, 0.74–1.21; I^2^ = 46%). By subgroup analysis, we found the high heterogeneity of local injection response did not originate from the type of BCG strain and suspected it might be related to the lack of a precise definition of a local injection reaction. In general, BCG vaccination is relatively safe.

The same result was concluded when Roborovski hamsters were subcutaneously vaccinated with BCG. Extensive damage to the pulmonary vasculature and significant levels of SARS-CoV-2 RNA were detected in the bone marrow of infected mice. Because off-target effects greatly relied on hematopoietic progenitor, which grew and developed in the bone marrow, SARS-CoV-2 was speculated to prevent off-target effects by damaging pulmonary vasculature and disseminating, which was a unique feature compared with other respiratory infections, such as influenza A virus (IAV) [16]. In conclusion, they speculated that due to the tissue tropism of COVID-19, BCG vaccination could not provide protection.

Similarly, Hilligan et al. demonstrated that BCG vaccination subcutaneously did not prevent SARS-CoV-2 infection nor severe COVID-19 in K18-hACE2 mice [33]. However, they found that intravenous injection of BCG can protect against lethal infection by reducing SCV2-induced tissue pathology, inflammatory cell recruitment, and excessive cytokine inflammatory responses [33,34]. In our study, BCG vaccination did not prevent severe COVID-19, possibly because intradermal BCG vaccination for humans is generally recommended except for treating bladder cancer. In contrast to intravenous vaccination in mice, human intradermal BCG vaccination is similar to subcutaneous vaccination in mice. 

Participants in all RCTs were vaccinated with COVID-19–specific vaccines at a later stage of the trial, and no differences were observed between the two groups. The current study showed BCG vaccination could enhance the efficacy of the SARS-CoV-2 vaccine utilizing boosting antibody and memory T-cell responses [35], but no differences in the rates of infection and hospitalization or mortality were found in the two groups. It has to be further investigated whether the strengthening of antibody and memory T-cell responses is powerful enough to prevent infection or reduce severe infection, and our study does not support BCG as a booster for the COVID-19 vaccine.

As opposed to what we found, a recent meta-analysis showed that BCG vaccination could protect against SARS-CoV-2 infection (OR, 0.61; 95% CI, 0.39–0.95; I^2^ = 31%) [36], perhaps because it included only (not merely) observational studies.

The study’s limitations are as follows:(1) The number of RCTs we included is small. Although more than forty clinical trials of BCG vaccination against COVID-19 have been registered, less than ten reports of these trials have been published [37]. For example, several clinical trials about the BCG vaccine to prevent COVID-19 for Health Care Workers are still ongoing in phase III trials [38,39,40,41]. Attention to the results of clinical trials in the coming years is needed to draw more accurate conclusions. (2) Only published studies were included because it is challenging to verify data in unpublished studies, especially when they have not undergone the rigorous peer review process. Although we discovered publication bias for the primary result, the trim-and-fill modification was conducted to guarantee the reliability of the results. (3) There was no information on the status of the measles vaccine (MV), oral polio vaccine (OPV), and measles-mumps-rubella (MMR), which may play the same role as BCG in “trained immunity” [42,43].

Regarding the strengths, in the meta-analysis, only RCTs were included, and the low heterogeneity ensured the credibility of the evidence. In addition, the sensitivity analysis performed on our primary outcome (the rate of infection) did not show noteworthy differences when deleting individual studies. Lastly, our findings support the 2020 WHO (World Health Organization) recommendation against using BCG vaccination to prevent COVID-19, which has important implications for avoiding unnecessary vaccination costs and a shortage of BCG vaccines [44].

## 5. Conclusions

Current findings do not support the assumption that BCG vaccination can protect against COVID-19 in terms of infection rate, admission to COVID-19-related hospitalization, admission to the ICU, and mortality. However, the number of RCTs we included is small, and the results of ongoing RCTs are important to validate this finding.

## Figures and Tables

**Table 1:** Study characteristics and participant demography.

Study	Year Published	Study Design	Participants Characteristics				Group		Timing of Follow-Up	Diagnosis of COVID-19
			Age (Mean, Year)	Mean Age (y)	Total Number (N)	Male (%)	Intervention (BCG Stain and Dosage)	Control		
Blossey [30]	2022	RCT multicenter	Older Adults	67.3	2025	52.9	BCG vaccination (VPM1002) (a genetically modified BCG) (2–8 × 105 colony forming units)	placebo	240 days	positive PCR
Sinha [27]	2022	RCT multicenter	Adults’ underlying medical conditions	43	495	52.1	0.1 mL BCG Moscow	Placebo	9 months	positive PCR
Tsilika [28]	2022	RCT Single center	Adults at risk	69	301	67.8	0.1 mL BCG Moscow	Placebo	6 months	positive PCR
Faustman [25]	2022	RCT Single center	Patients with type 1 diabetes	43.8	144	58.3	Multiple Tokyo 172 strain	Placebo	15 months	positive PCR and symptoms
Upton [29]	2022	RCT multicenter	Healthcare workers	39	1000	29.6	0.1 mL Danish strain 1331	Placebo	52 weeks	positive PCR
Doesschate [23]	2022	RCT multicenter	Healthcare workers	42	1511	25.7	0.1 mL Danish strain 1331	Placebo	26 weeks	positive PCR
Dos Anjos [24]	2022	RCT Single center	Healthcare workers	43	131	23.7	0.1 mL BCG Moscow	Unvaccinated	180 days	Positive PCR or rapid antigen test
Moorlag [26]	2022	RCT Single center	Older Adults	67	2014	52.5	0.1 mL Danish strain 1331	Placebo	12 months	positive PCR
Czajka [22]	2022	RCT multicenter	Healthcare workers	45	342	19.3	BCG vaccination in TST (-) participants Moreau Strain	Placebo in TST (-) participants	3 months	Positive PCR

BCG: Bacille Calmette-Guerin; TST: Tuberculin test results; RCT: a randomized controlled trial; PCR: polymerase chain reaction; COVID-19: Coronavirus disease 2019.

**Table 2:** GRADE assessment on the quality of the evidence.

Outcomes	No of Participants (Studies)	Quality of the Evidence (GRADE)	Relative Effect (95% CI)	Anticipated Absolute Effects	
				Risk with Control	Risk Difference with BCG Vaccination (95% CI)
**The rate of infection of COVID-19**	7963 (9 studies)	⊕⊕⊕⊝ **MODERATE** ^1^ due to publication bias	**OR 0.96** (0.82 to 1.13)	**Study population**	
				**89 per 1000**	**3 fewer per 1000** (from 15 fewer to 10 more)
				**Moderate**	
				**104 per 1000**	**4 fewer per 1000** (from 17 fewer to 12 more)
**The rate of COVID-19-related hospitalization**	7477 (7 studies)	⊕⊕⊕⊕ **HIGH**	**OR 0.66** (0.37 to 1.18)	**Study population**	
				**7 per 1000**	**3 fewer per 1000** (from 5 fewer to 1 more)
				**Moderate**	
				**8 per 1000**	**3 fewer per 1000** (from 5 fewer to 1 more)
**The rate of COVID-19-related admission to the ICU**	4534 (3 studies)	⊕⊕⊝⊝ **LOW** ^2^ due to imprecision	**OR 0.25** (0.05 to 1.18)	**Study population**	
				**3 per 1000**	**2 fewer per 1000** (from 3 fewer to 1 more)
				**Moderate**	
				**4 per 1000**	**3 fewer per 1000** (from 4 fewer to 1 more)
**The rate of COVID-19-related mortality**	5678 (5 studies)	⊕⊕⊝⊝ **LOW** ^3^ due to imprecision	**OR 0.64** (0.17 to 2.44)	**Study population**	
				**1 per 1000**	**1 fewer per 1000** (from 1 fewer to 2 more)
				**Moderate**	
				**1 per 1000**	**0 fewer per 1000** (from 1 fewer to 1 more)
**The rate of local injection response.**	4826 (4 studies)	⊕⊕⊝⊝ **LOW** ^4^ due to inconsistency	**OR 49.94** (14.08 to 177.2)	**Study population**	
				**33 per 1000**	**598 more per 1000** (from 292 more to 825 more)
				**Moderate**	
				**24 per 1000**	**527 more per 1000** (from 233 more to 789 more)
**The rate of local serious AEs.**	6477 (6 studies)	⊕⊕⊕⊕ **HIGH**	**OR 0.95** (0.74 to 1.21)	**Study population**	
				**42 per 1000**	**2 fewer per 1000** (from 10 fewer to 8 more)
				**Moderate**	
				**20 per 1000**	**1 fewer per 1000** (from 5 fewer to 4 more)

**CI:** Confidence interval; **OR:** Odds ratio; **ICU:** Intensive care units; **AEs:** Adverse events; **COVID-19:** Coronavirus disease 2019. GRADE Working Group grades of evidence **High quality:** Further research is very unlikely to change our confidence in the estimate of effect. **Moderate quality:** Further research is likely to have an important impact on our confidence in the estimate of effect and may change the estimate. **Low quality:** Further research is very likely to have an important impact on our confidence in the estimate of effect and is likely to change the estimate. **Very low quality:** We are very uncertain about the estimate. ^1^ Funnel plot asymmetry and/or significant Egger’s test (*p* = 0.017). ^2^ The number of events is small. ^3^ The number of events is small. ^4^ The heterogeneity is high (I^2^ = 92%).

## Data Availability

The datasets used and/or analyzed during this investigation are available upon reasonable request from the corresponding author.

## Supplementary Materials

The following supporting information can be downloaded at: https://www.mdpi.com/article/10.3390/jcm12031154/s1, Appendix S1: Search strategy; Appendix S2 Figure SA1: Subgroup analysis for the incidence of COVID-19 between the BCG vaccination group and the control group based on BCG strain; Figure SA2: Subgroup analysis for the incidence of local injection response between the BCG vaccination group and the control group based on BCG strain; Figure SA3: Publication bias analysis of the (a) COVID-19-related hospitalization; (b) COVID-19-related mortality; (c) serious AEs.
