# Supplementary material for: Efficacy of BCG Vaccination against COVID-19: Systematic Review and Meta-Analysis of Randomized Controlled Trials
Source: J Clin Med. 2023 Feb 1;12(3):1154. doi: 10.3390/jcm12031154 (PMC9917948; doi:10.3390/jcm12031154)
Supplement: Supplementary file 1 [file jcm-12-01154-s001.zip › jcm-2112541-supplementary.pdf]

Supplementary Material

Appendix S1. Search strategy

PubMed (496items)

no.	Search strategy
#1	"COVID-19"[Mesh]
#2	"COVID-19"[TW] OR "COVID 19"[TW] OR "COVID-19 Virus Disease"[TW] OR "COVID 19 Virus Disease"[TW] OR "COVID-19 Virus Diseases"[TW] OR "Disease, COVID-19 Virus"[TW] OR "Virus Disease, COVID-19"[TW] OR "COVID-19 Virus Infection"[TW] OR "COVID 19 Virus Infection"[TW] OR "COVID-19 Virus Infections"[TW] OR "Infection, COVID-19 Virus"[TW] OR "Virus Infection, COVID-19"[TW] OR "2019-nCoV Infection"[TW] OR "2019 nCoV Infection"[TW] OR "2019-nCoV Infections"[TW] OR "Infection, 2019-nCoV"[TW] OR "Coronavirus Disease-19"[TW] OR "Coronavirus Disease 19"[TW] OR "2019-nCoV Disease"[TW] OR "2019 nCoV Disease"[TW] OR "2019-nCoV Diseases"[TW] OR "Disease, 2019-nCoV"[TW] OR "COVID19"[TW] OR "Coronavirus Disease 2019"[TW] OR "Disease 2019, Coronavirus"[TW] OR "SARS Coronavirus 2 Infection"[TW] OR "SARS-CoV-2 Infection"[TW] OR "Infection, SARS-CoV-2"[TW] OR "SARS CoV 2 Infection"[TW] OR "SARS-CoV-2 Infections"[TW] OR "COVID-19 Pandemic"[TW] OR "COVID 19 Pandemic"[TW] OR "COVID-19 Pandemics"[TW] OR "Pandemic, COVID-19"[TW] OR "2019 Novel Coronavirus Disease"[TW] OR "2019 Novel Coronavirus Infection"[TW] OR "coronavirus disease 2"[TW] OR "coronavirus disease 2019 pneumonia"[TW] OR "coronavirus infection 2019"[TW] OR "COVID"[TW] OR "COVID 19 induced pneumonia"[TW] OR "COVID 2019"[TW] OR "COVID-19 induced pneumonia"[TW] OR "COVID-19 pneumonia"[TW] OR "nCoV 2019 disease"[TW] OR "nCoV 2019 infection"[TW] OR "paucisymptomatic coronavirus disease 2019"[TW] OR "SARS coronavirus 2 pneumonia"[TW] OR "SARSCoV2 disease"[TW] OR "SARS-CoV2 disease"[TW] OR "SARS-CoV-2 disease"[TW] OR "SARSCoV2 infection"[TW] OR "SARS-CoV2 infection"[TW] OR "SARS-CoV-2 pneumonia"[TW] OR "severe acute respiratory syndrome 2"[TW] OR "severe acute respiratory syndrome 2 pneumonia"[TW] OR "severe acute respiratory syndrome coronavirus 2 infection"[TW] OR "severe acute respiratory syndrome coronavirus 2019 infection"[TW] OR "severe acute respiratory syndrome CoV-2 infection"[TW] OR "Wuhan coronavirus disease"[TW] OR "Wuhan coronavirus infection"[TW] OR "2019 novel coronavirus epidemic"[TW] OR "new coronavirus

	pneumonia"[TW] OR "novel coronavirus 2019 disease"[TW] OR "novel coronavirus 2019 infection"[TW] OR "novel coronavirus disease 2019"[TW] OR "novel coronavirus infected pneumonia"[TW] OR "novel coronavirus infection 2019"[TW] OR "novel coronavirus pneumonia"[TW] OR "2019nCoV"[TW] OR "19nCoV"[TW] OR "COVID19*"[TW] OR "SARSCOV-2"[TW] OR "SARSCOV2"[TW] OR "corona virus 2"[TW] OR "Wuhan"[TW] OR "Hubei"[TW] OR "new coronavirus"[TW] OR "novel coronavirus"[TW] OR "novel corona virus"[TW] OR "novel CoV"[TW]
#3	"SARS-CoV-2"[Mesh]
#4	"SARS-CoV-2"[TW] OR "Coronavirus Disease 2019 Virus"[TW] OR "Wuhan Seafood Market Pneumonia Virus"[TW] OR "SARS-CoV-2 Virus"[TW] OR "SARS CoV 2 Virus"[TW] OR "SARS-CoV-2 Viruses"[TW] OR "Virus, SARS-CoV-2"[TW] OR "2019-nCoV"[TW] OR "COVID-19 Virus"[TW] OR "COVID 19 Virus"[TW] OR "COVID-19 Viruses"[TW] OR "Virus, COVID-19"[TW] OR "Wuhan Coronavirus"[TW] OR "Coronavirus, Wuhan"[TW] OR "SARS Coronavirus 2"[TW] OR "Coronavirus 2, SARS"[TW] OR "Severe Acute Respiratory Syndrome Coronavirus 2"[TW] OR "2019 Novel Coronavirus"[TW] OR "2019 Novel Coronaviruses"[TW] OR "Coronavirus, 2019 Novel"[TW] OR "Novel Coronavirus, 2019"[TW] OR "2019 nCOV"[TW] OR "2019 severe acute respiratory syndrome coronavirus 2"[TW] OR "HCoV-19"[TW] OR "Human coronavirus 2019"[TW] OR "nCoV-2019"[TW] OR "SARS2 (virus)"[TW] OR "SARS-related coronavirus 2"[TW] OR "Sever acute respiratory syndrome coronavirus 2"[TW] OR "Severe acute respiratory coronavirus 2"[TW] OR "Severe acute respiratory syndorme coronavirus 2"[TW] OR "severe acute respiratory syndrome 2 virus"[TW] OR "severe acute respiratory syndrome corona virus 2"[TW] OR "severe acute respiratory syndrome coronavirus 2019"[TW] OR "Severe acute respiratory syndrome coronoavirus 2"[TW] OR "Severe acute respiratory syndrome coronvirus 2"[TW] OR "severe acute respiratory syndrome CoV-2 virus"[TW] OR "Severe acute respiratory syndrome related coronavirus 2"[TW] OR "Severe acute respiratory syndrome virus 2"[TW] OR "Severe acute respiratoy syndrome coronavirus 2"[TW] OR "2019 new coronavirus"[TW] OR "novel 2019 coronavirus"[TW] OR "novel coronavirus-19"[TW]
#5	#1 OR #2 OR #3 OR #4
#6	"BCG Vaccine"[Mesh]
#7	"Mycobacterium bovis"[Mesh]
#8	"Vaccine, BCG"[TW] OR "Bacillus Calmette Guerin Vaccine"[TW] OR "Calmette’s Vaccine"[TW] OR "Calmette Vaccine"[TW] OR "Calmettes Vaccine"[TW] OR "Vaccine, Calmette’s"[TW] OR "Calmette Guerin Bacillus Vaccine"[TW] OR "BCG"[TW] OR " Calmette-Guerin

	Bacillus"[TW] OR "Bacilli Calmette Guerin"[TW] OR "Bacillus Calmette Guerin"[TW] OR "Bacille Calmette Guerin"[TW] OR "Bacilli Calmette Gerin"[TW] OR "Mycobacterium bovis"[TW] OR "BCG Vaccine"[TW] OR "Bacille Calmette-Guérin"[TW] OR "Bacille Calmette Guérin"[TW] OR "bacillus Calmette-Guérin vaccine"[TW] OR "Bacille Calmette Guérin Vaccine"[TW]
#9	#6 OR #7 OR #8
#10	#5 AND #9

Web of Science (579items)

no.	Search strategy
#1	TS=(COVID-19)
#2	AB=("COVID-19" OR "COVID 19" OR "COVID-19 Virus Disease" OR "COVID 19 Virus Disease" OR "COVID-19 Virus Diseases" OR "Disease, COVID-19 Virus" OR "Virus Disease, COVID-19" OR "COVID-19 Virus Infection" OR "COVID 19 Virus Infection" OR "COVID-19 Virus Infections" OR "Infection, COVID-19 Virus" OR "Virus Infection, COVID-19" OR "2019-nCoV Infection" OR "2019 nCoV Infection" OR "2019-nCoV Infections" OR "Infection, 2019-nCoV" OR "Coronavirus Disease-19" OR "Coronavirus Disease 19" OR "2019-nCoV Disease" OR "2019 nCoV Disease" OR "2019-nCoV Diseases" OR "Disease, 2019-nCoV" OR "COVID19" OR "Coronavirus Disease 2019" OR "Disease 2019, Coronavirus" OR "SARS Coronavirus 2 Infection" OR "SARS-CoV-2 Infection" OR "Infection, SARS-CoV-2" OR "SARS CoV 2 Infection" OR "SARS-CoV-2 Infections" OR "COVID-19 Pandemic" OR "COVID 19 Pandemic" OR "COVID-19 Pandemics" OR "Pandemic, COVID-19" OR "2019 Novel Coronavirus Disease" OR "2019 Novel Coronavirus Infection" OR "coronavirus disease 2" OR "coronavirus disease 2019 pneumonia" OR "coronavirus infection 2019" OR "COVID" OR "COVID 19 induced pneumonia" OR "COVID 2019" OR "COVID-19 induced pneumonia" OR "COVID-19 pneumonia" OR "nCoV 2019 disease" OR "nCoV 2019 infection" OR "paucisymptomatic coronavirus disease 2019" OR "SARS coronavirus 2 pneumonia" OR "SARSCoV2 disease" OR "SARS-CoV2 disease" OR "SARS-CoV-2 disease" OR "SARSCoV2 infection" OR "SARS-CoV2 infection" OR "SARS-CoV-2 pneumonia" OR "severe acute respiratory syndrome 2" OR "severe acute respiratory syndrome 2 pneumonia" OR "severe acute respiratory syndrome coronavirus 2 infection" OR "severe acute respiratory syndrome coronavirus 2019 infection" OR "severe acute respiratory syndrome CoV-2 infection" OR "Wuhan coronavirus disease" OR "Wuhan coronavirus infection" OR "2019 novel coronavirus epidemic" OR "new coronavirus pneumonia" OR "novel

	coronavirus 2019 disease" OR "novel coronavirus 2019 infection" OR "novel coronavirus disease 2019" OR "novel coronavirus infected pneumonia" OR "novel coronavirus infection 2019" OR "novel coronavirus pneumonia" OR "2019nCoV" OR "19nCoV" OR "COVID19*" OR "SARSCOV-2" OR "SARSCOV2" OR "corona virus 2" OR "Wuhan" OR "Hubei" OR "new coronavirus" OR "novel coronavirus" OR "novel corona virus" OR "novel CoV")
#3	TS=(SARS-CoV-2)
#4	AB=("SARS-CoV-2" OR "Coronavirus Disease 2019 Virus" OR "Wuhan Seafood Market Pneumonia Virus" OR "SARS-CoV-2 Virus" OR "SARS CoV 2 Virus" OR "SARS-CoV-2 Viruses" OR "Virus, SARS-CoV-2" OR "2019-nCoV" OR "COVID-19 Virus" OR "COVID 19 Virus" OR "COVID-19 Viruses" OR "Virus, COVID-19" OR "Wuhan Coronavirus" OR "Coronavirus, Wuhan" OR "SARS Coronavirus 2" OR "Coronavirus 2, SARS" OR "Severe Acute Respiratory Syndrome Coronavirus 2" OR "2019 Novel Coronavirus" OR "2019 Novel Coronaviruses" OR "Coronavirus, 2019 Novel" OR "Novel Coronavirus, 2019" OR "2019 nCOV" OR "2019 severe acute respiratory syndrome coronavirus 2" OR "HCoV-19" OR "Human coronavirus 2019" OR "nCoV-2019" OR "SARS2 (virus)" OR "SARS-related coronavirus 2" OR "Sever acute respiratory syndrome coronavirus 2" OR "Severe acute respiratory coronavirus 2" OR "Severe acute respiratory syndorme coronavirus 2" OR "severe acute respiratory syndrome 2 virus" OR "severe acute respiratory syndrome corona virus 2" OR "severe acute respiratory syndrome coronavirus 2019" OR "Severe acute respiratory syndrome coronoavirus 2" OR "Severe acute respiratory syndrome coronvirus 2" OR "severe acute respiratory syndrome CoV-2 virus" OR "Severe acute respiratory syndrome related coronavirus 2" OR "Severe acute respiratory syndrome virus 2" OR "Severe acute respiratoy syndrome coronavirus 2" OR "2019 new coronavirus" OR "novel 2019 coronavirus" OR "novel coronavirus-19")
#5	#1 OR #2 OR #3 OR #4
#6	TS=("BCG Vaccine")
#7	TS=("Mycobacterium bovis")
#8	AB=("Vaccine, BCG" OR "Bacillus Calmette Guerin Vaccine" OR "Calmette’s Vaccine" OR "Calmette Vaccine" OR "Calmettes Vaccine" OR "Vaccine, Calmette’s" OR "Calmette Guerin Bacillus Vaccine" OR "BCG" OR " Calmette-Guerin Bacillus" OR "Bacilli Calmette Guerin" OR "Bacillus Calmette Guerin" OR "Bacille Calmette Guerin" OR "Bacilli Calmette Gerin" OR "Mycobacterium bovis" OR "BCG Vaccine" OR "Bacille Calmette-Guérin" OR "Bacille Calmette Guérin" OR "bacillus Calmette-Guérin vaccine" OR "Bacille Calmette Guérin Vaccine")

#9	#6 OR #7 OR #8
#10	#5 AND #9

Ovid-EMBASE (556 items)

no.	Search strategy
#1	'coronavirus disease 2019'/exp
#2	'covid-19':ti,ab,kw OR 'covid 19':ti,ab,kw OR 'covid-19 virus disease':ti,ab,kw OR 'covid 19 virus disease':ti,ab,kw OR 'covid-19 virus diseases':ti,ab,kw OR 'disease, covid-19 virus':ti,ab,kw OR 'virus disease, covid-19':ti,ab,kw OR 'covid-19 virus infection':ti,ab,kw OR 'covid 19 virus infection':ti,ab,kw OR 'covid-19 virus infections':ti,ab,kw OR 'infection, covid-19 virus':ti,ab,kw OR 'virus infection, covid-19':ti,ab,kw OR '2019-ncov infection':ti,ab,kw OR '2019 ncov infection':ti,ab,kw OR '2019-ncov infections':ti,ab,kw OR 'infection, 2019-ncov':ti,ab,kw OR 'coronavirus disease-19':ti,ab,kw OR 'coronavirus disease 19':ti,ab,kw OR '2019-ncov disease':ti,ab,kw OR '2019 ncov disease':ti,ab,kw OR '2019-ncov diseases':ti,ab,kw OR 'disease, 2019-ncov':ti,ab,kw OR 'covid19':ti,ab,kw OR 'coronavirus disease 2019':ti,ab,kw OR 'disease 2019, coronavirus':ti,ab,kw OR 'sars coronavirus 2 infection':ti,ab,kw OR 'sars-cov-2 infection':ti,ab,kw OR 'infection, sars-cov-2':ti,ab,kw OR 'sars cov 2 infection':ti,ab,kw OR 'sars-cov-2 infections':ti,ab,kw OR 'covid-19 pandemic':ti,ab,kw OR 'covid 19 pandemic':ti,ab,kw OR 'covid-19 pandemics':ti,ab,kw OR 'pandemic, covid-19':ti,ab,kw OR '2019 novel coronavirus disease':ti,ab,kw OR '2019 novel coronavirus infection':ti,ab,kw OR 'coronavirus disease 2':ti,ab,kw OR 'coronavirus disease 2019 pneumonia':ti,ab,kw OR 'coronavirus infection 2019':ti,ab,kw OR 'covid':ti,ab,kw OR 'covid 19 induced pneumonia':ti,ab,kw OR 'covid 2019':ti,ab,kw OR 'covid-19 induced pneumonia':ti,ab,kw OR 'covid-19 pneumonia':ti,ab,kw OR 'ncov 2019 disease':ti,ab,kw OR 'ncov 2019 infection':ti,ab,kw OR 'paucisymptomatic coronavirus disease 2019':ti,ab,kw OR 'sars coronavirus 2 pneumonia':ti,ab,kw OR 'sarscov2 disease':ti,ab,kw OR 'sars-cov2 disease':ti,ab,kw OR 'sars-cov-2 disease':ti,ab,kw OR 'sarscov2 infection':ti,ab,kw OR 'sars-cov2 infection':ti,ab,kw OR 'sars-cov-2 pneumonia':ti,ab,kw OR 'severe acute respiratory syndrome 2':ti,ab,kw OR 'severe acute respiratory syndrome 2 pneumonia':ti,ab,kw OR 'severe acute respiratory syndrome coronavirus 2 infection':ti,ab,kw OR 'severe acute respiratory syndrome coronavirus 2019 infection':ti,ab,kw OR 'severe acute respiratory syndrome cov-2 infection':ti,ab,kw OR 'wuhan coronavirus disease':ti,ab,kw OR 'wuhan coronavirus

	infection':ti,ab,kw OR '2019 novel coronavirus epidemic':ti,ab,kw OR 'new coronavirus pneumonia':ti,ab,kw OR 'novel coronavirus 2019 disease':ti,ab,kw OR 'novel coronavirus 2019 infection':ti,ab,kw OR 'novel coronavirus disease 2019':ti,ab,kw OR 'novel coronavirus infected pneumonia':ti,ab,kw OR 'novel coronavirus infection 2019':ti,ab,kw OR 'novel coronavirus pneumonia':ti,ab,kw OR '2019ncov':ti,ab,kw OR '19ncov':ti,ab,kw OR "covid19$":ti,ab,kw OR 'sarscov-2':ti,ab,kw OR 'sarscov2':ti,ab,kw OR 'corona virus 2':ti,ab,kw OR 'wuhan':ti,ab,kw OR 'hubei':ti,ab,kw OR 'new coronavirus':ti,ab,kw OR 'novel coronavirus':ti,ab,kw OR 'novel corona virus':ti,ab,kw OR 'novel cov':ti,ab,kw
#3	'severe acute respiratory syndrome coronavirus 2'/exp
#4	('sars-cov-2':ti,ab,kw OR 'coronavirus disease 2019 virus':ti,ab,kw OR 'wuhan seafood market pneumonia virus':ti,ab,kw OR 'sars-cov-2 virus':ti,ab,kw OR 'sars cov 2 virus':ti,ab,kw OR 'sars-cov-2 viruses':ti,ab,kw OR 'virus, sars-cov-2':ti,ab,kw OR '2019-ncov':ti,ab,kw OR 'covid-19 virus':ti,ab,kw OR 'covid 19 virus':ti,ab,kw OR 'covid-19 viruses':ti,ab,kw OR 'virus, covid-19':ti,ab,kw OR 'wuhan coronavirus':ti,ab,kw OR 'coronavirus, wuhan':ti,ab,kw OR 'sars coronavirus 2':ti,ab,kw OR 'coronavirus 2, sars':ti,ab,kw OR 'severe acute respiratory syndrome coronavirus 2':ti,ab,kw OR '2019 novel coronavirus':ti,ab,kw OR '2019 novel coronaviruses':ti,ab,kw OR 'coronavirus, 2019 novel':ti,ab,kw OR 'novel coronavirus, 2019':ti,ab,kw OR '2019 ncov':ti,ab,kw OR '2019 severe acute respiratory syndrome coronavirus 2':ti,ab,kw OR 'hcov-19':ti,ab,kw OR 'human coronavirus 2019':ti,ab,kw OR 'ncov-2019':ti,ab,kw OR sars2:ti,ab,kw) AND virus:ti,ab,kw OR 'sars-related coronavirus 2':ti,ab,kw OR 'sever acute respiratory syndrome coronavirus 2':ti,ab,kw OR 'severe acute respiratory coronavirus 2':ti,ab,kw OR 'severe acute respiratory syndorme coronavirus 2':ti,ab,kw OR 'severe acute respiratory syndrome 2 virus':ti,ab,kw OR 'severe acute respiratory syndrome corona virus 2':ti,ab,kw OR 'severe acute respiratory syndrome coronavirus 2019':ti,ab,kw OR 'severe acute respiratory syndrome coronoavirus 2':ti,ab,kw OR 'severe acute respiratory syndrome coronvirus 2':ti,ab,kw OR 'severe acute respiratory syndrome cov-2 virus':ti,ab,kw OR 'severe acute respiratory syndrome related coronavirus 2':ti,ab,kw OR 'severe acute respiratory syndrome virus 2':ti,ab,kw OR 'severe acute respiratoy syndrome coronavirus 2':ti,ab,kw OR '2019 new coronavirus':ti,ab,kw OR 'novel 2019 coronavirus':ti,ab,kw OR 'novel coronavirus-19':ti,ab,kw
#5	#1 OR #2 OR #3 OR #4
#6	'mycobacterium bovis'/exp
#7	'mycobacterium bovis bcg'/exp

#8	bcg:ti,ab,kw OR ((bacille:ti,ab,kw OR bacili:ti,ab,kw OR bacilli:ti,ab,kw OR bacillus:ti,ab,kw) AND (calmette:ti,ab,kw OR calmet:ti,ab,kw) AND guerin:ti,ab,kw)
#9	#6 OR #7 OR #8
#10	#5 AND #9

Cochrane library (20 items)

no.	Search strategy
#1	[mh "COVID-19"]
#2	"SARS-CoV-2":ti,ab,kw OR "Coronavirus Disease 2019 Virus":ti,ab,kw OR "Wuhan Seafood Market Pneumonia Virus":ti,ab,kw OR "SARS-CoV-2 Virus":ti,ab,kw OR "SARS CoV 2 Virus":ti,ab,kw OR "SARS-CoV-2 Viruses":ti,ab,kw OR "Virus, SARS-CoV-2":ti,ab,kw OR "2019-nCoV":ti,ab,kw OR "COVID-19 Virus":ti,ab,kw OR "COVID 19 Virus":ti,ab,kw OR "COVID-19 Viruses":ti,ab,kw OR "Virus, COVID-19":ti,ab,kw OR "Wuhan Coronavirus":ti,ab,kw OR "Coronavirus, Wuhan":ti,ab,kw OR "SARS Coronavirus 2":ti,ab,kw OR "Coronavirus 2, SARS":ti,ab,kw OR "Severe Acute Respiratory Syndrome Coronavirus 2":ti,ab,kw OR "2019 Novel Coronavirus":ti,ab,kw OR "2019 Novel Coronaviruses":ti,ab,kw OR "Coronavirus, 2019 Novel":ti,ab,kw OR "Novel Coronavirus, 2019":ti,ab,kw OR "2019 nCOV":ti,ab,kw OR "2019 severe acute respiratory syndrome coronavirus 2":ti,ab,kw OR "HCoV-19":ti,ab,kw OR "Human coronavirus 2019":ti,ab,kw OR "nCoV-2019":ti,ab,kw OR "SARS2 (virus)":ti,ab,kw OR "SARS-related coronavirus 2":ti,ab,kw OR "Sever acute respiratory syndrome coronavirus 2":ti,ab,kw OR "Severe acute respiratory coronavirus 2":ti,ab,kw OR "Severe acute respiratory syndorme coronavirus 2":ti,ab,kw OR "severe acute respiratory syndrome 2 virus":ti,ab,kw OR "severe acute respiratory syndrome corona virus 2":ti,ab,kw OR "severe acute respiratory syndrome coronavirus 2019":ti,ab,kw OR "Severe acute respiratory syndrome coronoavirus 2":ti,ab,kw OR "Severe acute respiratory syndrome coronvirus 2":ti,ab,kw OR "severe acute respiratory syndrome CoV-2 virus":ti,ab,kw OR "Severe acute respiratory syndrome related coronavirus 2":ti,ab,kw OR "Severe acute respiratory syndrome virus 2":ti,ab,kw OR "Severe acute respiratoy syndrome coronavirus 2":ti,ab,kw OR "2019 new coronavirus":ti,ab,kw OR "novel 2019 coronavirus":ti,ab,kw OR "novel coronavirus-19":ti,ab,kw
#3	[mh "SARS-CoV-2"]
#4	"COVID-19":ti,ab,kw OR "COVID 19":ti,ab,kw OR "COVID-19 Virus Disease":ti,ab,kw OR "COVID 19 Virus Disease":ti,ab,kw

	OR "COVID-19 Virus Diseases":ti,ab,kw OR "Disease, COVID-19 Virus":ti,ab,kw OR "Virus Disease, COVID-19":ti,ab,kw OR "COVID-19 Virus Infection":ti,ab,kw OR "COVID 19 Virus Infection":ti,ab,kw OR "COVID-19 Virus Infections":ti,ab,kw OR "Infection, COVID-19 Virus":ti,ab,kw OR "Virus Infection, COVID-19":ti,ab,kw OR "2019-nCoV Infection":ti,ab,kw OR "2019 nCoV Infection":ti,ab,kw OR "2019-nCoV Infections":ti,ab,kw OR "Infection, 2019-nCoV":ti,ab,kw OR "Coronavirus Disease-19":ti,ab,kw OR "Coronavirus Disease 19":ti,ab,kw OR "2019-nCoV Disease":ti,ab,kw OR "2019 nCoV Disease":ti,ab,kw OR "2019-nCoV Diseases":ti,ab,kw OR "Disease, 2019-nCoV":ti,ab,kw OR "COVID19":ti,ab,kw OR "Coronavirus Disease 2019":ti,ab,kw OR "Disease 2019, Coronavirus":ti,ab,kw OR "SARS Coronavirus 2 Infection":ti,ab,kw OR "SARS-CoV-2 Infection":ti,ab,kw OR "Infection, SARS-CoV-2":ti,ab,kw OR "SARS CoV 2 Infection":ti,ab,kw OR "SARS-CoV-2 Infections":ti,ab,kw OR "COVID-19 Pandemic":ti,ab,kw OR "COVID 19 Pandemic":ti,ab,kw OR "COVID-19 Pandemics":ti,ab,kw OR "Pandemic, COVID-19":ti,ab,kw OR "2019 Novel Coronavirus Disease":ti,ab,kw OR "2019 Novel Coronavirus Infection":ti,ab,kw OR "coronavirus disease 2":ti,ab,kw OR "coronavirus disease 2019 pneumonia":ti,ab,kw OR "coronavirus infection 2019":ti,ab,kw OR "COVID":ti,ab,kw OR "COVID 19 induced pneumonia":ti,ab,kw OR "COVID 2019":ti,ab,kw OR "COVID-19 induced pneumonia":ti,ab,kw OR "COVID-19 pneumonia":ti,ab,kw OR "nCoV 2019 disease":ti,ab,kw OR "nCoV 2019 infection":ti,ab,kw OR "paucisymptomatic coronavirus disease 2019":ti,ab,kw OR "SARS coronavirus 2 pneumonia":ti,ab,kw OR "SARSCoV2 disease":ti,ab,kw OR "SARS-CoV2 disease":ti,ab,kw OR "SARS-CoV-2 disease":ti,ab,kw OR "SARSCoV2 infection":ti,ab,kw OR "SARS-CoV2 infection":ti,ab,kw OR "SARS-CoV-2 pneumonia":ti,ab,kw OR "severe acute respiratory syndrome 2":ti,ab,kw OR "severe acute respiratory syndrome 2 pneumonia":ti,ab,kw OR "severe acute respiratory syndrome coronavirus 2 infection":ti,ab,kw OR "severe acute respiratory syndrome coronavirus 2019 infection":ti,ab,kw OR "severe acute respiratory syndrome CoV-2 infection":ti,ab,kw OR "Wuhan coronavirus disease":ti,ab,kw OR "Wuhan coronavirus infection":ti,ab,kw OR "2019 novel coronavirus epidemic":ti,ab,kw OR "new coronavirus pneumonia":ti,ab,kw OR "novel coronavirus 2019 disease":ti,ab,kw OR "novel coronavirus 2019 infection":ti,ab,kw OR "novel coronavirus disease 2019":ti,ab,kw OR "novel

	coronavirus infected pneumonia":ti,ab,kw OR "novel coronavirus infection 2019":ti,ab,kw OR "novel coronavirus pneumonia":ti,ab,kw OR "2019nCoV":ti,ab,kw OR "19nCoV":ti,ab,kw OR "COVID19*":ti,ab,kw OR "SARSCOV-2":ti,ab,kw OR "SARSCOV2":ti,ab,kw OR "corona virus 2":ti,ab,kw OR "Wuhan":ti,ab,kw OR "Hubei":ti,ab,kw OR "new coronavirus":ti,ab,kw OR "novel coronavirus":ti,ab,kw OR "novel corona virus":ti,ab,kw OR "novel CoV":ti,ab,kw
#5	#1 OR #2 OR #3 OR #4
#6	[mh "BCG Vaccine"]
#7	[mh "Mycobacterium Bovis"]
#8	#6 OR #7
#9	#5 AND #8

Appendix S2

Study or Subgroup	BCG Vaccination Events	BCG Vaccination Total	Control Events	Control Total	Weight	Odds Ratio M-H, Fixed, 95% CI
**1.2.1 BCG Moscow**						
Dos Anjos 2022	8	64	11	67	3.8%	0.73 [0.27, 1.94]
Sinha 2022	18	246	17	249	6.4%	1.08 [0.54, 2.14]
Tsilika 2022	2	148	7	153	2.8%	0.29 [0.06, 1.40]
**Subtotal (95% CI)**		**458**		**469**	**13.0%**	**0.81 [0.48, 1.35]**
Total events	28		35			
Heterogeneity: Chi² = 2.37, df = 2 (P = 0.31); I² = 15%						
Test for overall effect: Z = 0.82 (P = 0.41)						
**1.2.2 Danish strain 1331**						
Doesschate 2022	102	753	108	758	38.0%	0.94 [0.70, 1.26]
Moorlag 2022	51	1008	48	1006	18.6%	1.06 [0.71, 1.59]
Upton 2022	99	500	93	500	30.4%	1.08 [0.79, 1.48]
**Subtotal (95% CI)**		**2261**		**2264**	**87.0%**	**1.02 [0.84, 1.23]**
Total events	252		249			
Heterogeneity: Chi² = 0.45, df = 2 (P = 0.80); I² = 0%						
Test for overall effect: Z = 0.17 (P = 0.86)						
**Total (95% CI)**		**2719**		**2733**	**100.0%**	**0.99 [0.83, 1.18]**
Total events	280		284			
Heterogeneity: Chi² = 3.31, df = 5 (P = 0.65); I² = 0%						
Test for overall effect: Z = 0.12 (P = 0.91)						
Test for subgroup differences: Chi² = 0.68, df = 1 (P = 0.41), I² = 0%						

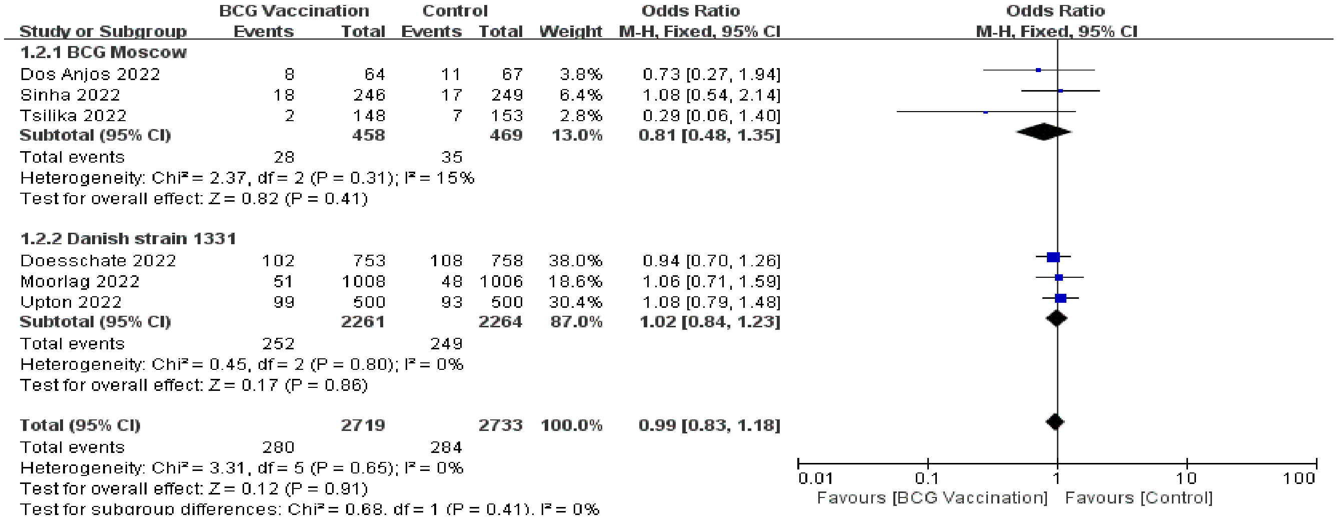

Figure SA1: Subgroup analysis for the incidence of COVID-19 between the BCG vaccination group and the control group based on BCG strain.

Study or Subgroup	BCG Vaccination Events	BCG Vaccination Total	Control Events	Control Total	Weight	Odds Ratio M-H, Fixed, 95% CI
**5.3.1 BCG Moscow**						
Dos Anjos 2022	59	753	3	758	14.6%	21.40 [6.68, 68.56]
Tsilika 2022	7	148	1	153	5.0%	7.55 [0.92, 62.10]
**Subtotal (95% CI)**		**901**		**911**	**19.5%**	**17.88 [6.49, 49.24]**
Total events	66		4			
Heterogeneity: Chi² = 0.73, df = 1 (P = 0.39); I² = 0%						
Test for overall effect: Z = 5.58 (P < 0.00001)						
**5.3.2 Danish strain 1331**						
Moorlag 2022	758	1008	55	1006	72.2%	52.43 [38.56, 71.27]
Upton 2022	463	500	21	500	8.2%	285.43 [164.59, 494.97]
**Subtotal (95% CI)**		**1508**		**1506**	**80.5%**	**76.23 [58.42, 99.48]**
Total events	1221		76			
Heterogeneity: Chi² = 27.80, df = 1 (P < 0.00001); I² = 96%						
Test for overall effect: Z = 31.91 (P < 0.00001)						
**Total (95% CI)**		**2409**		**2417**	**100.0%**	**64.83 [49.38, 85.12]**
Total events	1287		80			
Heterogeneity: Chi² = 37.16, df = 3 (P < 0.00001); I² = 92%						
Test for overall effect: Z = 30.03 (P < 0.00001)						
Test for subgroup differences: Chi² = 7.36, df = 1 (P = 0.007), I² = 86.4%						

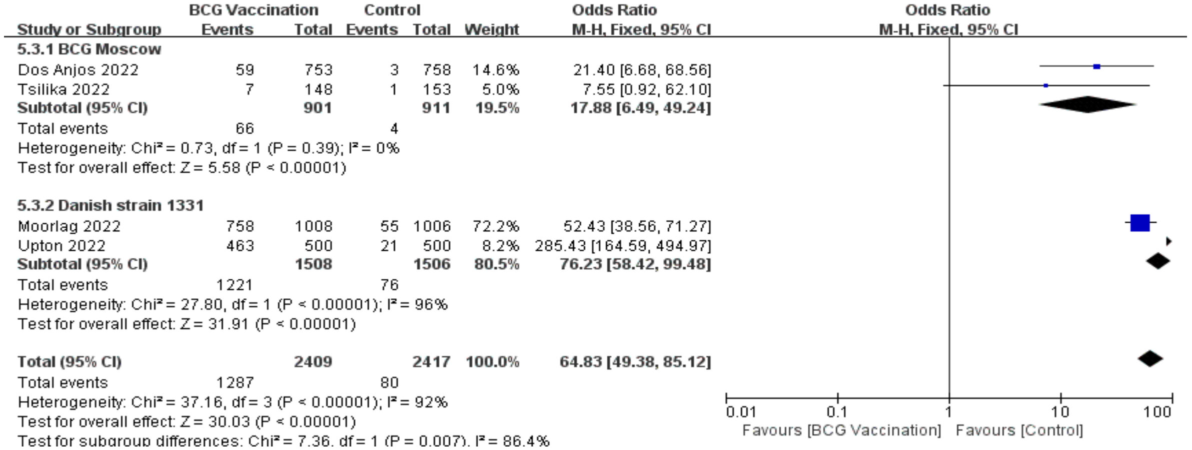

Figure SA2: Subgroup analysis for the incidence of local injection response between the BCG vaccination group and the control group based on BCG strain.

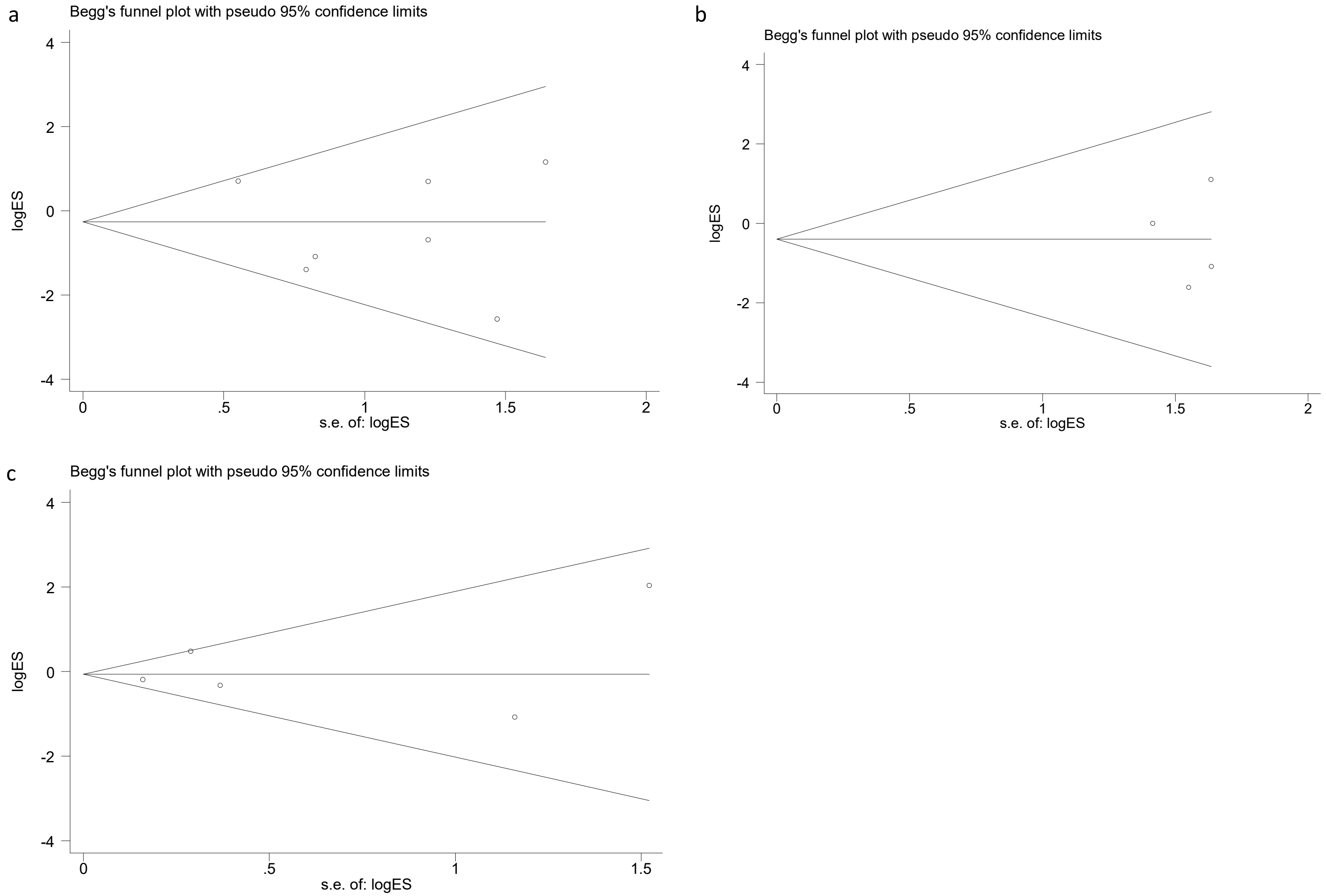

Figure SA3: Publication bias analysis of the (a)COVID-19-related hospitalization; (b)COVID-19-related mortality; (c)serious AEs.
